# Supplementary material for: Nitrogen-Doped Porous Carbon Derived from Coal for High-Performance Dual-Carbon Lithium-Ion Capacitors
Source: Nanomaterials (Basel). 2023 Sep 9;13(18):2525. doi: 10.3390/nano13182525 (PMC10536825; doi:10.3390/nano13182525)
Supplement: Supplementary file 1 [file nanomaterials-13-02525-s001.zip › nanomaterials-2594322-supplementary.pdf]

# Supporting Information

# Nitrogen-Doped Porous Carbon Derived from Coal for High-Performance Dual-Carbon Lithium-Ion Capacitors

**Jiangmin Jiang [1,*], Qianqian Shen [2,*], Ziyu Chen [1] and Shijing Wang [3]**

[1] Jiangsu Province Engineering Laboratory of High Efficient Energy Storage Technology and Equipments, School of Materials Science and Physics, China University of Mining and Technology, Xuzhou 221116, China

[2] School of Materials Science and Engineering, Zhejiang University, Hangzhou 310027, China

[3] Tsinghua Shenzhen International Graduate School, Tsinghua University, Shenzhen 518055, China

* Correspondence: jiangmin326@163.com (J.J.); 12326050@zju.edu.cn (Q.S.)

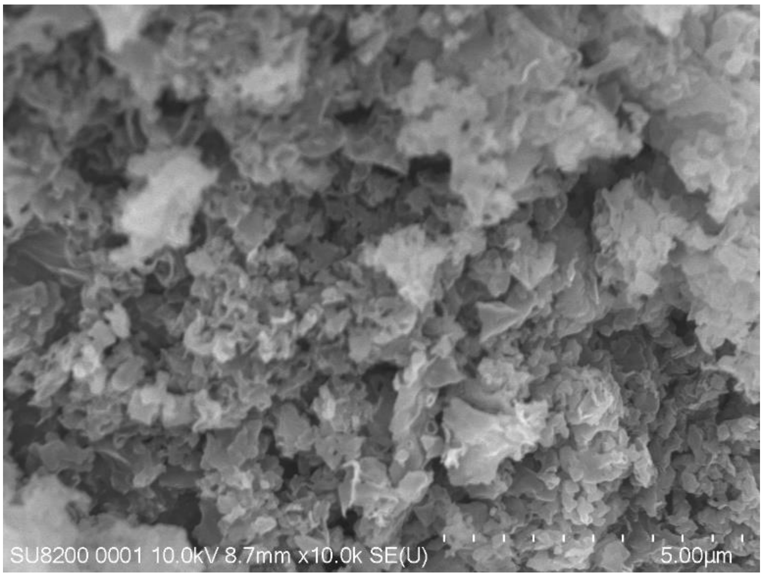


**Figure S1**. SEM image of the g-$C_3N_4$.

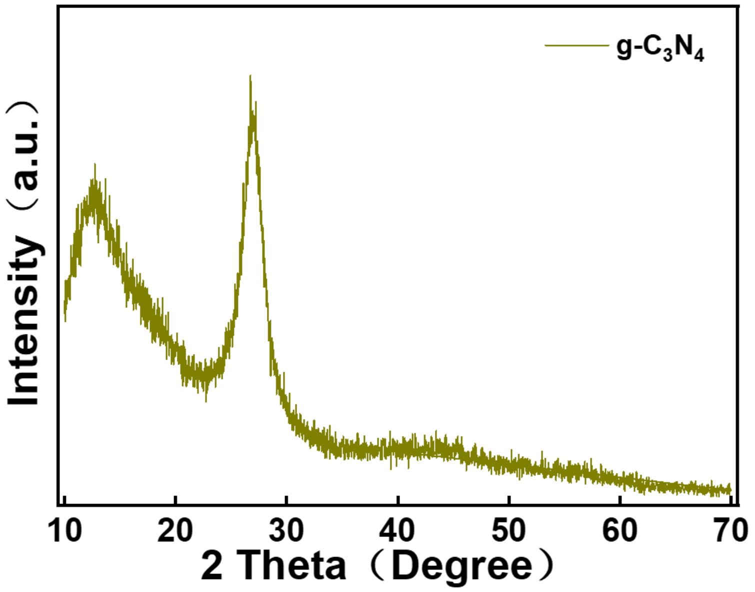


**Figure S2**. XRD pattern of the g-$C_3N_4$.

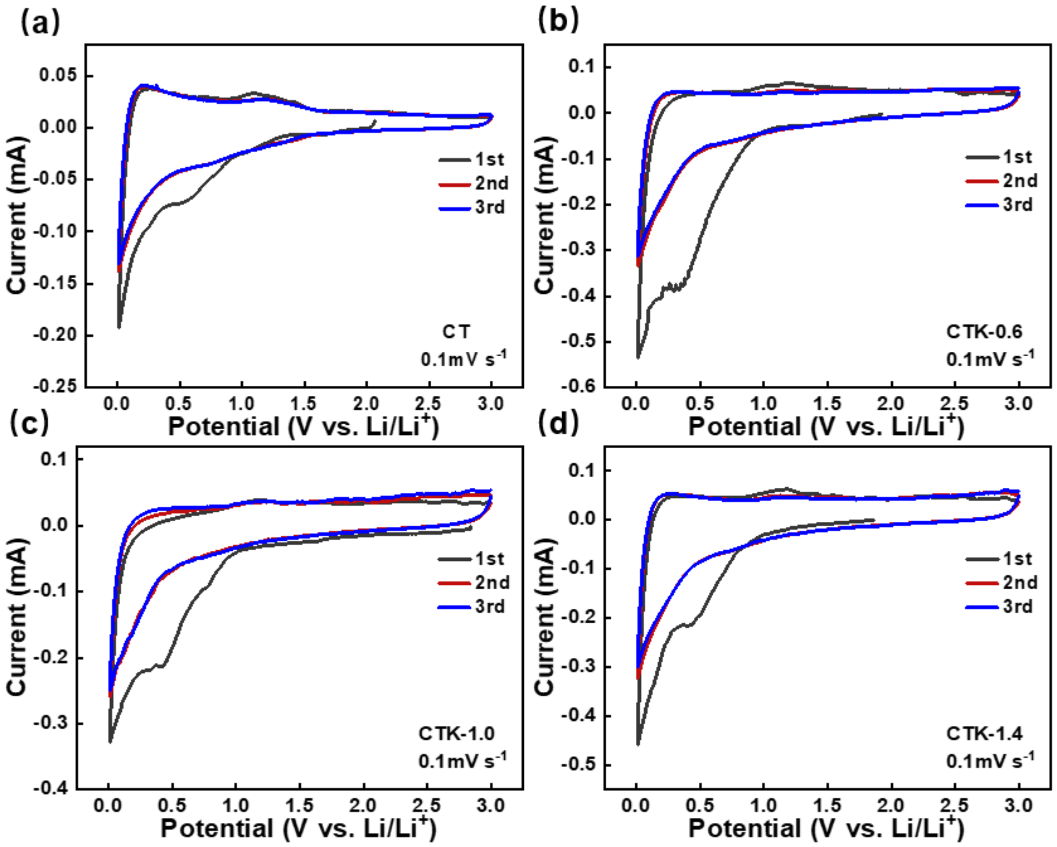


**Figure S3**. CV curves of the (a) CT, (b) CTK-0.6, (c) CTK-1.0, and (d) CTK-1.4 with the first three cycles at the scanning rate of 0.1mV $s^{-1}$.

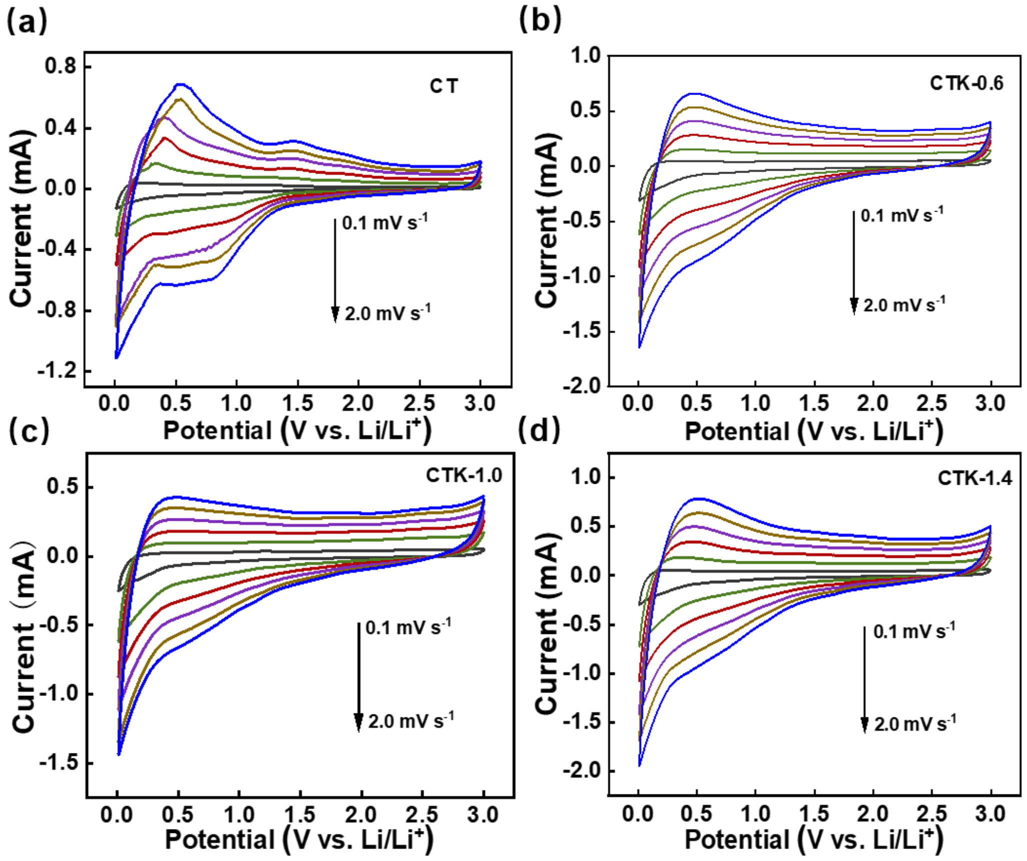


**Figure S4**. CV curves of the (a) CT, (b) CTK-0.6, (c) CTK-1.0, and (d) CTK-1.4 at different scanning rates from 0.1~2.0 mV $s^{-1}$.

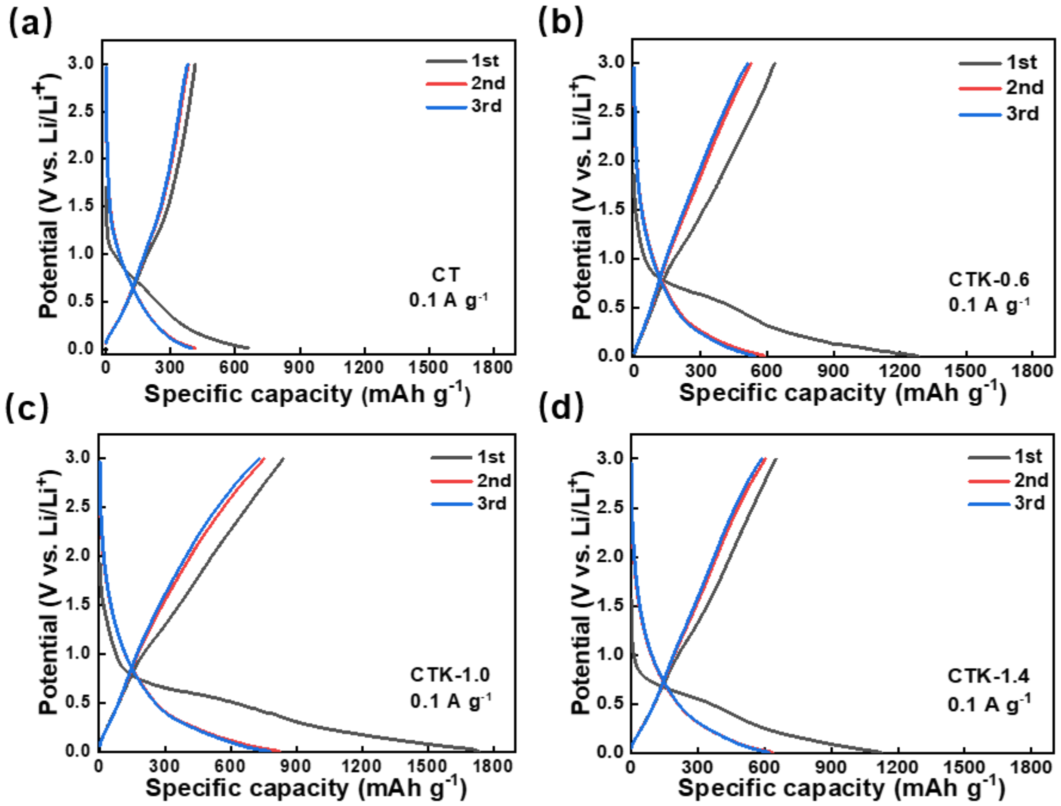


**Figure S5.** The constant charge-discharge curves of (a) CT, (b) CTK-0.6, (c) CTK-1.0, and (d) CTK-1.4.

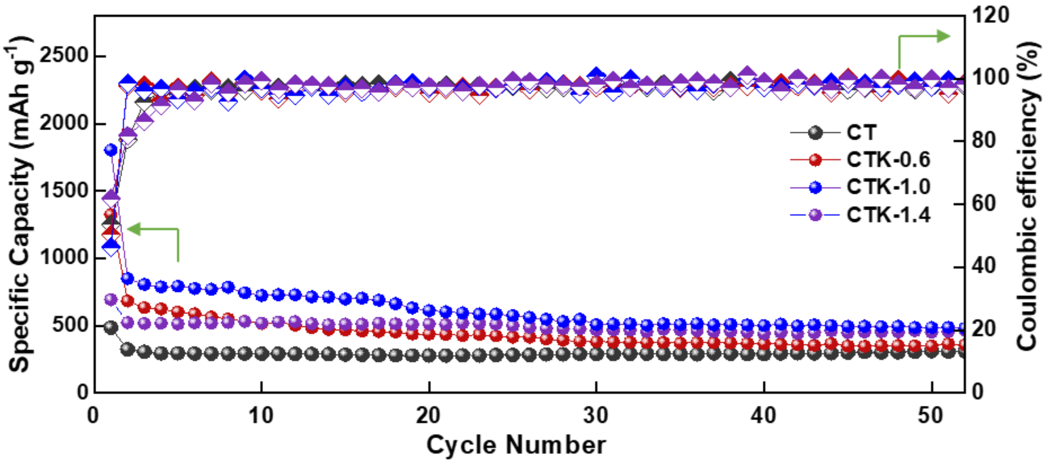


**Figure S6.** The cycling performance of CT and CNPCs at a current density of 100 mA $g^{-1}$.

**Table S1.** Elements content obtained from the XPS data of all samples.

| Sample | C (at.%) | N (at.%) | O (at.%) | S (at.%) |
|---|---|---|---|---|
| CTK-0.6 | 90.85 | 1.26 | 7.78 | 0.10 |
| CTK-1.0 | 91.74 | 3.03 | 5.02 | 0.11 |
| CTK-1.4 | 82.41 | 4.34 | 13.12 | 0.13 |

**Table S2.** Pore structures of all samples.

| Sample | SSA ($m^2$ $g^{-1}$) | Pore volume ($cm^3$ $g^{-1}$) |
|---|---|---|
| CT | ~0 | 0.000013 |
| CTK-0.6 | 1479.3 | 0.61 |
| CTK-1.0 | 1673.5 | 0.77 |
| CTK-1.4 | 848.8 | 0.39 |

**Table S3.** Performance comparison of DC-LICs (CTK-1.0//CTK-1.0) with other LICs.

| LIC systems | Energy density and corresponding power density | Voltage window (V) | Refs. |
|---|---|---|---|
| GNS-13//AC | 112 Wh $kg^{-1}$ @30 W $kg^{-1}$<br>10.9 Wh $kg^{-1}$ @19.6 kW $kg^{-1}$ | 2.0~4.0 | [1] |
| 100-LTO-G-600C//AC | 52Wh $kg^{-1}$ @225 W $kg^{-1}$<br>35 Wh $kg^{-1}$ @14.4 kW $kg^{-1}$ | 1.5~3.0 | [2] |
| T-$Nb_2O_5$@C//MSP-20 | 63 Wh $kg^{-1}$ @70 W $kg^{-1}$<br>5 Wh $kg^{-1}$ @16.5 kW $kg^{-1}$ | 1.0~3.5 | [3] |
| $MnNb_2O_6$@rGO//AC | 118.2 Wh $kg^{-1}$ @0.1 kW $kg^{-1}$<br>68.5 Wh $kg^{-1}$ @8 kW $kg^{-1}$ | 0~4.0 | [4] |
| LDAC//3DC@LTSO | 115.3 Wh $kg^{-1}$ @163.5 W $kg^{-1}$<br>60 Wh $kg^{-1}$ @6.560 kW $kg^{-1}$ | 0.5~4.0 | [5] |
| GC1100//SFAC-2 | 104 Wh $kg^{-1}$ @143 W $kg^{-1}$<br>32 Wh $kg^{-1}$ @6.628 kW $kg^{-1}$ | 2.0~4.0 | [6] |
| PHPNC//TiC | 101.5 Wh $kg^{-1}$ @450 W $kg^{-1}$<br>23.4 Wh $kg^{-1}$ @67.5 kW $kg^{-1}$ | 0~4.5 | [7] |

| | | | |
|---|---|---|---|
| $Ti_3C_2T_x$/CNT//AC | 67 Wh kg$^{-1}$ @258 W kg$^{-1}$<br>19 Wh kg$^{-1}$ @5.8 kW kg$^{-1}$ | 1.0~4.0 | [8] |
| PGCs//AC | $E_{max}$: 83.7 Wh kg$^{-1}$<br>$P_{max}$: 6.5 kW kg$^{-1}$ | 2.0~4.0 | [9] |
| Commercialized LICs | E: 20~50 Wh kg$^{-1}$<br>P: 30 kW kg$^{-1}$ | 2.0~4.0 | / |
| **CTK-1.0//CTK-1.0** | **137.2 Wh kg$^{-1}$ @ 410 W kg$^{-1}$**<br>**71 Wh kg$^{-1}$ @20.5 kW kg$^{-1}$** | **0.1~4.2** | **This work** |

**References**


1. Li, G.; Huang, Y.; Yin, Z.; Guo, H.; Liu, Y.; Cheng, H.; Wud, Y.; Ji, X.; Wang, J. Defective synergy of 2D graphitic carbon nanosheets promotes lithium-ion capacitors performance. *Energy Storage Mater.* **2020**, *24*, 304-11.
2. Wang, G.; Lu, C.; Zhang, X.; Wan, B.; Liu, H.; Xia, M.; Gou, H.; Xin, G.; Lian, J.; Zhang, Y. Toward ultrafast lithium ion capacitors: A novel atomic layer deposition seeded preparation of $Li_4Ti_5O_{12}$/graphene anode. *Nano Energy* **2017**, *36*, 46-57.
3. Lim, E.; Jo, C.; Kim, H.; Kim, M.; Mun, Y.; Chun, J.; Ye, Y.; Hwang, J.; Ha, K.; Roh, K.; Kang, K.; Yoon, S.; Lee, J. Facile Synthesis of $Nb_2O_5$@Carbon Core-Shell Nanocrystals with Controlled Crystalline Structure for High-Power Anodes in Hybrid Supercapacitors. *ACS Nano* **2015**, *9*, 7497-505.
4. Zhang, X.; Zhang, J.; Kong, S.; Zhu, K.; Yan, J.; Ye, K.; Wang, G.; Cheng, K.; Zhou, L.; Cao, D. A novel calendula-like $MnNb_2O_6$ anchored on graphene sheet as high-performance intercalation pseudocapacitive anode for lithium-ion capacitors. *J. Mater. Chem. A* **2019**, *7*, 2855-63.
5. Jin, L.; Gong, R.; Zhang, W.; Xiang, Y.; Zheng, J.; Xiang, Z.; Zhang, C.; Xia, Y.; Zheng, J. Toward high energy-density and long cycling-lifespan lithium ion capacitors: a 3D carbon modified low-potential $Li_2TiSiO_5$ anode coupled with a lignin-derived activated carbon cathode. *J. Mater. Chem. A* **2019**, *7*, 8234-44.

6. Yang, Z.; Guo, H.; Li, X.; Wang, Z.; Wang, J.; Wang, Y.; Yan, Z.; Zhang, D. Graphitic carbon balanced between high plateau capacity and high rate capability for lithium ion capacitors. *J. Mater. Chem. A* **2017**, *5*, 15302-9.
7. Wang, H.; Zhang, Y.; Ang, H.; Zhang, Y.; Tan, H.; Zhang, Y.; Guo, Y.; Franklin, J.; Wu, X.; Srinivasan, M.; Fan, H.; Yan, Q. A High-Energy Lithium-Ion Capacitor by Integration of a 3D Interconnected Titanium Carbide Nanoparticle Chain Anode with a Pyridine-Derived Porous Nitrogen-Doped Carbon Cathode. *Adv. Funct. Mater.* **2016**, *26*, 3082-93.
8. Yu, P.; Cao, G.; Yi, S.; Zhang, X.; Li, C.; Sun, X.; Wang, K.; Ma, Y. Binder-free 2D titanium carbide (MXene)/carbon nanotube composites for high-performance lithium-ion capacitors. *Nanoscale* **2018**, *10*, 5906-13.
9. Li, G.; Yin, Z.; Guo, H.; Wang, Z.; Yan, G.; Yang, Z.; Liu, Y.; Ji, X.; Wang, J. Metalorganic Quantum Dots and Their Graphene-Like Derivative Porous Graphitic Carbon for Advanced Lithium-Ion Hybrid Supercapacitor. *Adv. Energy Mater.* **2019**, *9*, 1802878.
